# AI vs. MD: Benchmarking ChatGPT and Gemini for Complex Wound Management

**DOI:** 10.3390/jcm14248825

**Published:** 2025-12-13

**Authors:** Luca Corradini, Gianluca Marcaccini, Ishith Seth, Warren M. Rozen, Camilla Biagiotti, Roberto Cuomo, Francesco Ruben Giardino

**Affiliations:** 1 Plastic Surgery Unit, Department of Medicine, Surgery and Neuroscience, University of Siena, 53100 Siena, Italy; l.corradini@student.unisi.it (L.C.); roberto.cuomo@unisi.it (R.C.);; 2 Department of Plastic and Reconstructive Surgery, Frankston Hospital, Peninsula Health, Frankston, VIC 3199, Australiawarrenrozen@hotmail.com (W.M.R.); 3 Faculty of Medicine and Surgery, Peninsula Clinical School, Monash University, Frankston, VIC 3199, Australia; 4 Department of Civil and Industrial Engineering (DICI), University of Pisa, Largo Lucio Lazzarino, 56122 Pisa, Italy

**Keywords:** large language models, wound management, complex wounds, chronic wounds, ChatGPT, Gemini, teleconsultation, artificial intelligence

## Abstract

**Background:** The management of hard-to-heal wounds poses a major clinical challenge due to heterogeneous etiology and significant global healthcare costs (estimated at USD 148.64 billion in 2022). Large Language Models (LLMs), such as ChatGPT and Gemini, are emerging as potential decision-support tools. This study aimed to rigorously assess the accuracy and reliability of ChatGPT and Gemini in the visual description and initial therapeutic management of complex wounds based solely on clinical images. **Methods:** Twenty clinical images of complex wounds from diverse etiologies were independently analyzed by ChatGPT (version dated 15 October 2025) and Gemini (version dated 15 October 2025). The models were queried using two standardized, concise prompts. The AI responses were compared against a clinical gold standard established by the unanimous consensus of an expert panel of three plastic surgeons. **Results:** Statistical analysis showed no significant difference in overall performance between the two models and the expert consensus. Gemini achieved a slightly higher percentage of perfect agreement in management recommendations (75.0% vs. 60.0% for ChatGPT). Both LLMs demonstrated high proficiency in identifying the etiology of vascular lesions and recognizing critical “red flags,” such as signs of ischemia requiring urgent vascular assessment. Noted divergences included Gemini’s greater suspicion of potential neoplastic etiology and the models’ shared error in suggesting Negative Pressure Wound Therapy (NPWT) in a case potentially contraindicated by severe infection. **Conclusions:** LLMs, particularly ChatGPT and Gemini, demonstrate significant potential as decision-support systems and educational tools in wound care, offering rapid diagnosis and standardized initial management, especially in non-specialist settings. Instances of divergence in systemic treatments or in atypical presentations highlight the limitations of relying on image-based reasoning alone. Ultimately, LLMs serve as powerful, scalable assets that, under professional supervision, can enhance diagnostic speed and improve care pathways.

## 1. Introduction

Plastic and reconstructive surgeons are routinely confronted with the management of hard-to-heal wounds, a broad category encompassing chronic wounds characterized by delayed, arrested, or disrupted healing due to intrinsic and/or extrinsic factors acting on the patient or the wound itself [1]. Although several definitions have been proposed, no universally accepted temporal threshold currently exists beyond which a wound is unequivocally classified as chronic [2].

The etiology of these wounds is highly heterogeneous and, in many cases, multiple pathogenic mechanisms interact synergistically to promote their onset and hinder normal healing processes [3]. Chronic or hard-to-heal wounds are generally classified into two major groups [4,5]: typical wounds, which account for approximately 80% of all chronic lesions and include pressure, venous, arterial and diabetic foot ulcers, and atypical or rare wounds, such as chronic traumatic, dehiscent or infected surgical, neoplastic and radiation-induced ulcers and other less common entities. The absence of a universally accepted definition complicates efforts to estimate the global prevalence of chronic wounds [6]. However, recent analyses suggest that these conditions affect approximately 1–2% of the world’s population [7], with global healthcare expenditure for their management estimated at around USD 148.64 billion in 2022 [8].

Hard-to-heal wounds constitute a major clinical challenge due to frequent biofilm formation and the absence of standardized diagnostic tools capable of detecting early determinants of chronicity [9,10]. Therapeutic management is further complicated by the poor response to conventional treatments and the growing need for individualized, multimodal therapeutic strategies [11]. It is well established that the delayed initiation of targeted therapy increases the risk of infection, impaired healing, amputation, and escalating healthcare costs [8,12]. Accordingly, early and accurate diagnosis, combined with timely, patient-specific intervention, is essential to optimize healing outcomes and mitigate the clinical and economic burden associated with chronic wounds.

For more than two decades, the TIME framework [13,14] has been recognized as a structured and comprehensive approach to the management of hard-to-heal wounds. The acronym represents the four fundamental components on which the model is based: Tissue (removal of nonviable or necrotic tissue), Infection/Inflammation (identification and control of infection and inflammation), Moisture balance (maintenance of an optimal moisture level within the wound bed), and Edge (stimulation of epithelial edge advancement and wound margin migration). Subsequent adaptations have expanded the model to enhance therapeutic individualization. The TIME Clinical Decision Support Tool (TIME CDST), for instance, incorporates broader assessments of the patient’s overall clinical condition and systemic factors, in addition to local wound-bed parameters [15]. The modified TIME-H model further emphasizes the role of patient comorbidities (H—Health), integrating a scoring system designed to predict both the likelihood and timeframe of wound healing based not only on wound type, but also on patient-specific health status. Its prognostic accuracy ranges from approximately 63% in vascular-origin wounds to 88% in surgical wounds [16].

In this complex scenario, fueled by new therapeutic approaches and increasingly robust knowledge in wound management, Large Language Models (LLMs) are emerging as potentially transformative tools in clinical practice. Their capability to convert heterogeneous data into structured and actionable information, and to extract key elements from provided inputs to suggest an adequate management approach for complex wounds, has been shown to enhance the precision of wound assessments, facilitate early infection detection, and streamline clinical workflows [17]. When integrated with computer vision models (a term validated and used here), as demonstrated by tools like SkinGPT-4, LLMs have proven adept at identifying salient features within wound images, generating differential diagnoses, and proposing therapeutic strategies. This functionality is particularly applicable in remote triage pathways [18]. In routine clinical practice, these technologies could therefore support non-specialist personnel and nursing teams in the early identification of warning signs, suggest up-to-date or equivalent dressing options based on the latest evidence, reduce the time required to initiate appropriate therapy, and limit heterogeneity in initial decision-making [19,20]. Furthermore, LLMs applied in the field of complex wounds could serve as a training tool for junior plastic surgeons or less experienced practitioners, offering a sophisticated platform for diagnostic and therapeutic consultation and comparison [21,22].

These opportunities, however, are counterbalanced by significant safety and reliability limitations. LLMs remain vulnerable to “hallucination,” defined as the generation of plausible but clinically erroneous assertions [23,24]. Additionally, they risk amplifying biases already present in their training data. Errors of this nature can translate into misleading or potentially harmful recommendations, if not adequately controlled through the analysis of authoritative sources and human supervision. Recent studies have also highlighted the risk of “automation bias,” which is the tendency of clinicians to attribute excessive credibility to linguistically coherent, but not always correct AI-generated responses [25,26]. This underscores the need for cautious implementation strategies and systematic monitoring of errors, especially as non-specialists, who benefit most from decision support systems, are often the most susceptible to automation bias.

The currently available literature presents substantial gaps that limit the possibility of a fully informed clinical adoption of LLMs in complex wound care. A large portion of the available results is derived from optimized and synthetic datasets or benchmarks not representative of day-to-day practice. Crucially, there is a lack of studies that rigorously compare the therapeutic plans proposed by AI with those developed by independent expert panels [27]. Consequently, claims regarding the clinical utility of LLMs remain provisional and require extensive validation in representative real-world cohorts.

In view of the aforementioned limitations, the present study was meticulously designed to rigorously assess the accuracy and reliability of two prominent LLMs, ChatGPT and Gemini, in the descriptive analysis and management of complex wounds, based exclusively on a single clinical image. This approach replicates a realistic triage scenario, such as a remote teleconsultation, a community nursing assessment, or an initial emergency department evaluation, where contextual information regarding patient comorbidities and medical history is inherently limited. Furthermore, this setting directly addresses the models’ potential for educational application for trainees and less experienced healthcare providers, serving as a structured tool for decision support.

The primary objective of this study is to determine the degree of concordance between the initial therapeutic plans suggested by the AI and those formulated by an expert panel of plastic surgeons, in a clinically realistic context based solely on images of complex wounds, without access to other patient information. Secondary objectives include evaluating the descriptive accuracy of the models, their ability to identify clinically relevant elements (such as necrosis, maceration, or signs of infection), their decision safety, defined as the absence of potentially harmful recommendations, and the educational value of the responses for non-specialist healthcare personnel.

## 2. Materials and Methods

The study was conducted in accordance with the principles of the Declaration of Helsinki and current regulations governing scientific research and data protection. Since all wound images used were fully anonymized and obtained from publicly accessible sources, formal approval from an ethics committee was not required. All procedures followed international guidelines for observational research involving public domain visual material.

Clinical images of wounds of different etiologies and levels of complexity were selected to represent a broad spectrum of conditions, including traumatic, postoperative, infected, and ulcerative lesions. The images were obtained from the website https://www.medetec.co.uk/files/medetec-image-databases.html (accessed on 9 October 2025), which authorizes the use of its content for research and scientific dissemination purposes. Permission for image use was granted according to the site’s terms, and all images were processed in a completely anonymized form, with the removal of metadata and any identifiable elements.

Each image was analyzed by two large language models (LLMs): ChatGPT (version dated 15 October 2025) and Gemini (version dated 15 October 2025). Both models were queried independently, with no exchange of information or iterative regeneration, using two standardized prompts applied uniformly to all cases: Provide a brief, objective clinical description of the wound. Focus only on visible features and key clinical details (max 3 lines) and state the most appropriate wound dressing or basic management plan. Give only the essential recommendation without explanation (max 2 lines). For each image, each prompt was submitted once to each model and the first complete response was recorded, without repeating or regenerating the query, using the default web interface configuration.

These prompts, reported in full in the manuscript, were designed to obtain from each model a concise clinical description and an essential therapeutic recommendation, replicating a visual triage scenario without access to patient history or additional clinical data.

In parallel, a panel of 3 experienced plastic surgeons analyzed the same images, providing an objective clinical description and a corresponding management plan for each case. Their evaluations were discussed collectively until a unanimous consensus was reached, which was taken as the clinical reference standard for the study. The surgeons reviewed each case together in a structured consensus session, discussing any divergent opinions until a single unanimous diagnosis and initial management plan were agreed. For each AI–surgeon comparison, concordance was rated on a three-level ordinal scale, where 0 indicated a discordant (inaccurate or unsafe) response, 1 a partially concordant (incomplete or partially correct) response, and 2 a fully concordant response (clinically accurate and aligned with the expert consensus).

**Figure 1:**
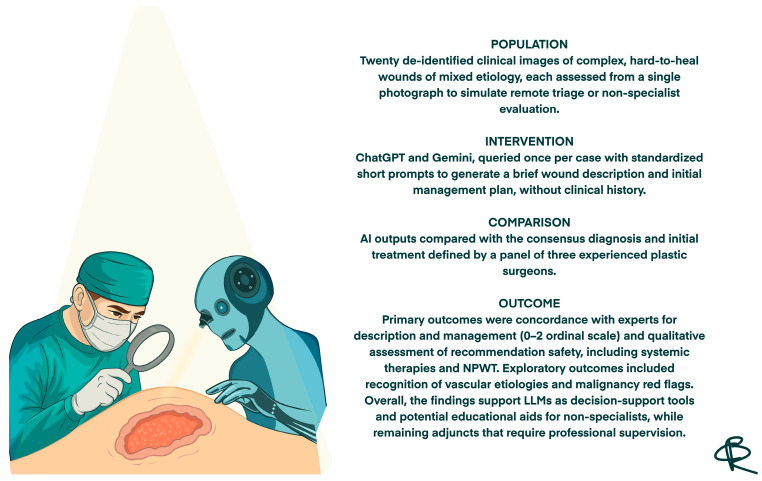
Structured summary of the study design and main outcomes based on the PICO framework.

In PICO terms, the Population consisted of de-identified clinical images of complex hard-to-heal wounds of different etiologies and severities; the Intervention corresponded to the assessments generated by the two large language models (ChatGPT and Gemini) in response to standardized prompts; the Comparator was the consensus diagnosis and initial management plan provided by a panel of experienced plastic surgeons; and the primary Outcomes were the degree of concordance between AI and experts for wound description and initial management, together with the safety of the recommendations (Figure 1).

## 3. Results

All 20 wound cases were processed by both models, and case-by-case descriptive and management outputs are summarized in Table 1. Table 2 reports the visual concordance matrix between each AI model and the surgeons’ consensus for wound description and initial management. As shown in Table 3, for wound description the mean concordance scores with the expert panel were 1.50 (SD 0.51) for ChatGPT and 1.55 (SD 0.69) for Gemini, with fully concordant descriptions in 10/20 (50.0%) and 13/20 (65.0%) cases, respectively. For initial management, mean scores were 1.60 (SD 0.50) for ChatGPT and 1.70 (SD 0.57) for Gemini, with fully concordant management in 12/20 (60.0%) and 15/20 (75.0%) cases; the proportion of fully concordant management decisions was significantly higher than 50% for Gemini (*p* = 0.041). Inter-model agreement, measured by Cohen’s κ, was 0.000 for wound descriptions and 0.255 for management. Overall performance scores (sum of description and management) were 3.10 (SD 0.64) for ChatGPT and 3.25 (SD 0.97) for Gemini (*p* = 0.388).

## 4. Discussion

Our study assessed the accuracy and clinical applicability of LLMs, ChatGPT and Gemini, in the visual description and therapeutic management of 20 hard-to-heal wounds, with each evaluated solely from a photographic image. Although statistical analysis did not reveal significant differences in overall performance compared with the gold standard, the qualitative assessment provides critical insights into how these technologies may be integrated into clinical practice. However, these non-significant results should not be interpreted as formal equivalence; with only 20 cases, the study is underpowered to detect modest but potentially relevant differences in performance and should therefore be regarded as exploratory.

A particularly noteworthy finding is the proficiency demonstrated by both models in identifying the etiology of vascular lesions. ChatGPT and Gemini consistently recognized the hallmark clinical features of venous ulcers, like edema, hyperpigmentation, anatomical location, and correctly recommended compression therapy as the cornerstone of management. Similarly, with specific reference to case 8, which presented critical limb ischaemia with dry necrosis, both models demonstrated high diagnostic sensitivity, achieving a perfect agreement (score 2) by appropriately advising urgent vascular evaluation and avoiding unsafe recommendations such as aggressive debridement. These findings align with a recent meta-analysis by Jimenez [28], which highlighted the capability of AI systems to correctly identify chronic wound etiology and propose appropriate treatment plans. This capacity for reliable aetiological discrimination represents a key strength and highlights the potential role of LLMs as first-line screening and decision-support tools, for example, in community-based nursing settings, as suggested by Pressmann and Liang [19,20], facilitating the prompt identification of patients requiring urgent medical management. From a practical perspective, these findings suggest that such tools could be piloted as structured aids for early wound triage in community and emergency settings, and as case-based learning resources that help trainees and non-specialist staff practise systematic description and initial management of complex wounds. 

The models diverged substantially in therapeutic planning. Overall, Gemini tended to produce more structured, sequential, and algorithmic management plans; yet this methodical approach was not without pitfalls in complex scenarios, as was evident in cases 3, 8, 13, and 18. By contrast, ChatGPT adopted a more conservative stance and adhered closely to prompt instructions, although it frequently conveyed unwarranted confidence and lacked the probabilistic nuance expected in scenarios with inherent diagnostic uncertainty. This pattern of rigid adherence by ChatGPT versus the broader elaborative freedom exhibited by Gemini aligns with literature, specifically the works of Salbas et al. on brain MRI sequences [29] and Sami et al. within pediatric radiology [30]. In our comparison, Gemini often appeared more attentive to the description and treatment of secondary lesions or the perilesional skin compared to ChatGPT. This discrepancy may stem from ChatGPT’s stricter adherence to the prompt, which likely constrained its field of analysis. Future studies should therefore explore alternative, less restrictive prompting strategies to assess whether differences in output length and structure reflect true reasoning performance or are primarily driven by prompt sensitivity.

An inconsistency was noted regarding systemic antibiotic therapy recommendations. Specifically, in case 2, ChatGPT failed to propose necessary antibiotic treatment. This omission represents a significant risk, particularly if we consider the potential future use of these systems for home-based wound management by non-professionals. However, this was not a systematic failure: the same model correctly suggested systemic antibiotics in other instances of suspected infection (case 5 and 9), aligning with previous findings by Nelson et al. [17] regarding the potential utility of AI in early infection detection. Notably, despite variability in systemic prescribing, both models consistently recommended antimicrobial dressings in suspected infection, demonstrating a reliable understanding of local infection control. 

Interestingly, both ChatGPT and Gemini erroneously proposed Negative Pressure Wound Therapy (NPWT) in the case of an infected abdominal wound dehiscence (case 10), failing to recognize the potential signs of severe infection which would contraindicate immediate NPWT without prior radical debridement. This error illustrates how a narrowly framed prompt focused on local wound management, without explicitly requesting systemic evaluation, can divert the model’s attention from critical priorities such as sepsis workup and urgent surgical re-exploration. Consequently, the models may have prioritized wound bed preparation over a more urgent and aggressive diagnostic and therapeutic pathway. This finding underscores the fundamental limitation of relying on LLMs for critical decision-making, caveat already emphasized by Aljindan in oculoplastics [31] and Gomez-Cabello in plastic surgery [32]: clinical judgment and systemic evaluation cannot be delegated to image-based reasoning alone. Beyond this example, the discrepancies summarized in Table 1 span a spectrum from benign variability in dressing choice, through conditionally unsafe delays or omissions in systemic therapy, to clearly unsafe recommendations, and were qualitatively considered in our safety assessment.

Another significant shortcoming observed is the difficulty both models exhibited in recognizing the etiology of a lesion when it appeared in an atypical anatomical location. This was evident in case 12 (supposed pressure lesion on the lateral aspect of the first metatarsal head) and case 16 (supposed pressure ulcer on the mandibular area). This deficiency likely reflects the statistical nature of LLM training: when a pathology is strongly associated with a canonical anatomical site in the medical literature, the model may struggle to identify it when it occurs out of context. This finding aligns with evidence from dermatological AI research, where diagnostic accuracy significantly degrades when anatomical priors are missing or when lesions occur in sites historically underrepresented in training datasets, such as acral or mucosal regions [33,34]. This suggests a lack of true pathophysiological reasoning, relying instead on pattern matching that fails when the presentation is non-standard. More broadly, these errors likely reflect biases in the underlying training data distributions and highlight the need for curated, anatomically diverse wound image datasets when developing and deploying LLM-based tools for clinical use.

Despite these weaknesses, an additional strength, particularly for Gemini, warrants emphasis: its feature-extraction ability in atypical wounds. Gemini more readily suspected the potential neoplastic nature of certain complex wounds (e.g., breast ulceration and a scalp lesion) and correctly recommended diagnostic biopsy, whereas ChatGPT more often defaulted to standard wound-care protocols.

This study presents several limitations that must be acknowledged. First, the sample size of cases analyzed is relatively small and may not capture the full variability of AI responses across the full spectrum of hard-to-heal wounds, so the present study should be regarded as exploratory and hypothesis-generating. Second, the evaluation was primarily text-based; as visual inspection is the gold standard in plastic surgery, the inability to assess the true multimodal capabilities of the models limits the generalisability of our findings to real-world clinical practice [27]. In particular, relying on single still images without patient history, comorbidities, perfusion data or longitudinal evolution represents a methodological constraint, as real-world wound assessment is inherently multimodal. Additionally, the influence of prompt engineering must be considered: while the input instructions were designed to be targeted and adequate for eliciting comprehensive and non-dispersive answers, further optimization of the prompts could potentially alter the models’ performance. In this regard, a divergence in instruction adherence was also observed: only ChatGPT strictly complied with the limitations regarding response length, whereas Gemini consistently generated outputs exceeding the requested parameters.

More fundamentally, condensing complex wound assessment and triage decisions into very short, three-line prompts inevitably removes much of the clinical context that would normally guide expert judgment. This design likely biases the models toward simplified, pattern-based answers and may underestimate their potential performance in more realistic, context-rich settings. Future studies should therefore test multi-step conversational prompts and multimodal inputs that combine images with structured clinical data, to better approximate real-world clinical workflows. Furthermore, the rapid evolution of LLMs means that the versions evaluated may already have been superseded by newer iterations with different behaviors. Finally, the assessment of “correctness” relied on qualitative expert judgment without a double-blinded review, introducing an element of subjectivity. Future studies should therefore adopt independent, blinded expert ratings and report inter-rater reliability.

Future research should aim to expand upon the foundational findings presented here. While this study intentionally utilized widely accessible, free-to-use AI models to ensure reproducibility and simulate a realistic scenario applicable to daily practice, future investigations could extend this comparison to include other currently available general-purpose LLMs, as well as specialized AI systems specifically trained on wound care datasets. Furthermore, to better approximate complex clinical decision-making, subsequent studies should move beyond isolated visual assessment by integrating complete patient history and clinical metadata alongside wound images; this would verify whether added context improves safety and therapeutic precision. It would also be valuable to increase the statistical robustness of these evaluations by expanding the sample size of lesions analyzed and comparing the AI outputs against multiple distinct panels of experts, to better account for variability in human clinical judgment. Another crucial aspect to explore is the longitudinal assessment of wound management. Monitoring the same lesion over time would allow researchers to verify how AI recommendations adapt to the physical evolution of the wound and whether the models can effectively guide the continuation of therapy, taking into account the outcomes of previously indicated dressings. Finally, looking towards a more distant horizon, prospective studies integrating these tools into real-world clinical workflows, such as community nursing, would be the ultimate test of their practical utility, although the implementation of such trials remains a complex challenge at this stage.

## 5. Conclusions

The findings of this study provide compelling evidence regarding the reliability and clinical potential of Large Language Models in the field of wound care. ChatGPT and Gemini demonstrated a remarkable degree of concordance with the expert panel, exhibiting high sensitivity in the descriptive analysis of lesions and the recognition of critical “red flags,” such as signs of ischemia or infection. These tools can play a supportive role not only in clinical practice but also as potential adjuncts in medical education, for example, as an interactive training platform where students and residents can compare their descriptions and management plans with AI-generated outputs, although this educational role was not formally assessed in the present study.

Furthermore, the ability of these models to rapidly generate coherent management plans highlights their value as effective decision-support systems, particularly in non-specialist settings where they can facilitate the timely triage of complex patients. While instances of divergence regarding systemic treatments, such as in the case of abdominal dehiscence, indicate that visual analysis benefits significantly from the integration of clinical history, they do not diminish the utility of the instrument. Ultimately, LLMs represent a powerful asset for the modern plastic surgeon: they should remain an adjunct rather than a replacement for clinical judgment, and particular caution is needed when non-specialists rely on their suggestions, given the risks of over-reliance and automation bias. When used under professional supervision and within appropriate institutional and regulatory frameworks, they can offer a scalable, always-on resource that helps enhance diagnostic speed, standardize care, and support continuous medical training.

## Figures and Tables

**Table 1:** Comparison between AI-generated wound assessments and surgeons’ evaluations. Each case was analyzed by ChatGPT and Gemini using two standardized prompts: Provide a brief, objective clinical description of the wound. Focus only on visible features and key clinical details (max 3 lines). State the most appropriate wound dressing or basic management plan. Give only the essential recommendation without explanation (max 3 lines). The table presents the descriptions and management suggestions generated by both models alongside the corresponding surgeon’s evaluation and recommended treatment.

Case ID	Clinical Case	ChatGPT—Wound Description	ChatGPT—Suggested Management	Gemini—Wound Description	Gemini—Suggested Management	Surgeons’ Consensus on Wound Description	Surgeons’ Consensus on Recommended Management
1	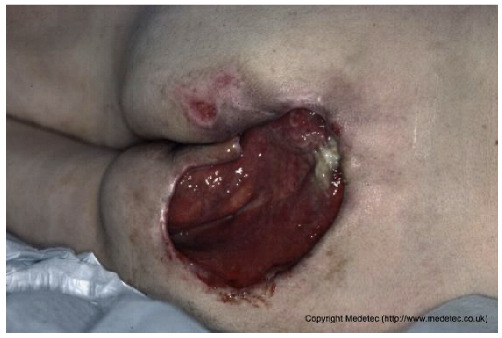	Large sacral pressure ulcer with exposed subcutaneous tissue and muscle, showing moist red granulation tissue mixed with areas of fibrinous slough. The wound edges are irregular and macerated, with surrounding erythema and a smaller superficial lesion superiorly.	Alginate or hydrofiber dressing with secondary foam cover; initiate pressure relief, wound debridement and/or NPWT as needed.	The image displays a large, deep wound located over the gluteal/sacral region. The wound bed appears to be filled with exposed, deep red, viable tissue (likely muscle/fat) and exhibits significant tissue loss, consistent with a full-thickness injury. There is minimal surrounding erythema, and some slough/exudate is visible in the wound base.	Wound management should involve debridement if necessary, followed by a deeply absorptive dressing (e.g., foam or alginate) or negative pressure wound therapy (NPWT) to promote granulation and manage high exudate, along with infection control.	Extensive stage IV pressure ulcer with muscular exposure involving the sacral and left gluteal regions. The wound bed is exudative, showing evident granulation tissue with a limited area of fibrin deposition in the cranial portion. Wound edges are irregular, undermined, and slightly macerated. The perilesional skin appears edematous. On the right sacral area, a smaller stage III pressure ulcer is also present.	Surgical debridement of the limited area of fibrin deposition, protease-modulating matrix and foam; alternatively, NPWT. Pressure-relieving devices and alternative patient repositioning.
2	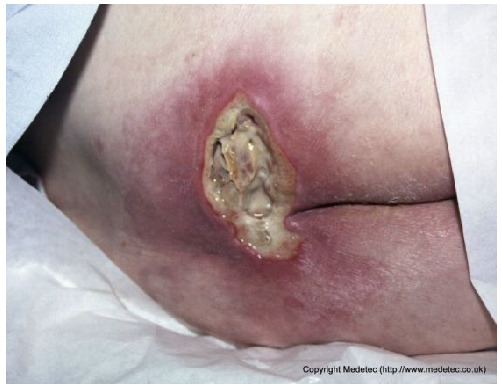	Deep sacral ulcer with central necrotic slough and purulent exudate, surrounded by erythematous and inflamed skin. The wound margins are irregular and macerated, with signs of local infection.	Debridement of necrotic tissue, application of hydrofiber or alginate dressing with antimicrobial properties, and consistent pressure off-loading.	The image shows a wound in the sacral/gluteal region with surrounding intense erythema and inflammation. The wound bed contains a significant amount of thick, yellow-brown slough/necrotic tissue and purulent exudate. The wound appears to be a full-thickness injury with clear signs of local infection/inflammation (cellulitis).	Aggressive management for presumed infection is required, including systemic antibiotics and urgent surgical or sharp debridement to remove the necrotic tissue. Topical management should include an antimicrobial dressing (e.g., silver) or antiseptic cleanser/gel.	Extensive pressure ulcer of probable stage III–IV etiology, located in the coccygeal region. The wound bed is exudative, with abundant moist necrotic tissue and slough. The presence of an infectious component cannot be excluded. Wound margins are irregular, exhibiting an erythematous peripheral halo. The perilesional skin shows extensive and marked erythema, likely of infectious or inflammatory origin.	Perform a wound culture swab. Surgical debridement of the necrotic tissue and slough down to a well-vascularized tissue; hydrofiber or antimicrobial dressing (i.e., silver containing dressing) and foam. Evaluate the administration of systemic antibiotics Pressure-relieving devices and alternative patient repositioning.
3	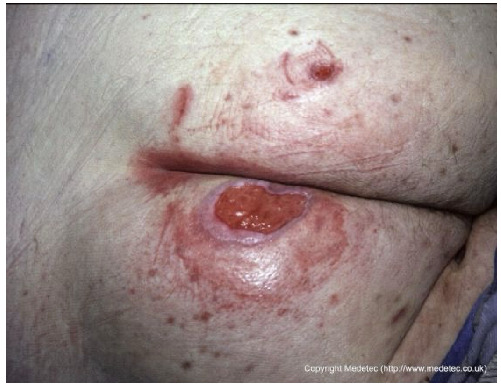	Shallow sacral ulcer with clean red granulation tissue and minimal exudate, surrounded by erythematous, mildly macerated skin. Wound edges are well defined without visible necrosis.	Hydrocolloid or foam dressing to maintain a moist environment, with regular repositioning and skin protection from friction and moisture.	The image shows a partial-thickness wound over the gluteal/sacral region with a clean, granulating red base and no visible slough or necrosis. The periwound skin is intensely erythematous, macerated/denuded, and excoriated, suggesting significant moisture-associated skin damage (MASD) or incontinence-associated dermatitis (IAD). The primary wound is small and shallow with clear margins.	Manage periwound moisture-associated skin damage (MASD) with a skin barrier cream/ointment (or liquid film). Apply a hydrocolloid or thin foam dressing to the main wound to maintain a moist environment for healing.	Right sacral ulcer, likely stage III pressure-related, oval in shape with an approximate diameter of 5 cm. The wound bed appears clean, granulating, and with minimal clear exudate. Wound margins are well-defined and display a pinkish-violet hue, suggestive of re-epithelialization. The perilesional skin is erythematous, with evidence of intertrigo along the intergluteal fold. A similar ulcer, though smaller in size, is present in the left sacral region.	Thin hydrocolloid or low-adherence polyurethane foam dressing. Skin barrier cream on perilesional skin. Pressure-relieving devices and alternative patient repositioning.
4	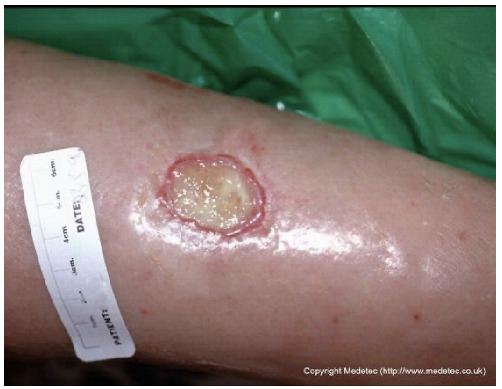	Oval-shaped ulcer on the lower leg with a central area of yellow fibrinous slough, surrounded by erythematous and mildly edematous skin. The wound edges are well defined and slightly raised.	Hydrofiber or alginate dressing with gentle debridement and compression therapy if venous etiology is confirmed.	The image shows a small, shallow, circular wound on the lower leg with a well-defined rolled/epibole edge. The wound base contains a moderate amount of pale yellow, translucent slough/fibrin, with minimal surrounding erythema. The periwound skin is intact but appears glossy and slightly edematous.	Manage the epibole with gentle debridement/abrasion of the wound edges. Apply a hydrogel to the sloughy base under a semi-occlusive film or hydrocolloid to promote autolytic debridement and maintain moisture.	Oval-shaped ulcer of probable venous etiology, approximately 2 cm in diameter, located on the anterior aspect of the leg. The wound bed is exudative and covered with slough. Wound margins are well-defined, irregular, and erythematous. The perilesional skin appears mildly edematous.	Surgical debridement or mechanical abrasion of the slough down to a well-vascularized tissue; hydrofiber and low-adherence polyurethane foam dressing; evaluate compression therapy.
5	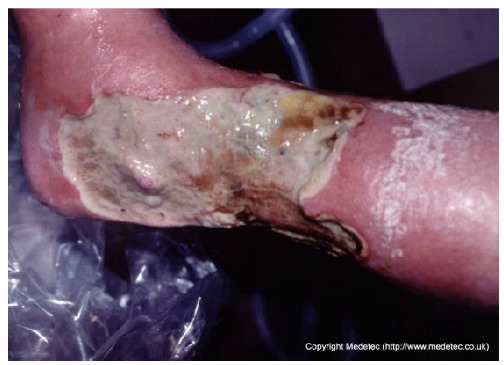	Extensive ulcer on the lower leg with thick yellow-green necrotic slough and areas of black eschar, surrounded by erythematous and edematous skin. The wound surface appears moist with signs of infection.	Sharp or enzymatic debridement, application of antimicrobial alginate or silver dressing, and initiation of systemic antibiotics if infection is confirmed.	The image displays a large, irregular wound on the lower leg/ankle, primarily covered by thick, pale yellow slough and central black eschar/necrosis. The wound depth appears superficial/partial-thickness. The surrounding skin is hyper-pigmented, significantly erythematous, and possibly weeping, suggesting chronic venous insufficiency and acute inflammation.	Prioritize compression therapy for underlying venous insufficiency. Debridement is necessary for the slough and eschar; apply a hydrogel or cadexomer iodine under a highly absorptive dressing (e.g., foam) to manage exudate and promote autolytic debridement.	Large ulcer of probable venous etiology, located on the medial and distal aspects of the right leg. The lesion shows full-thickness tissue loss, with a necrotic and slough-covered exudative wound bed. A dry eschar is present in the proximal-posterior portion. An infectious component is considered plausible. Wound margins are irregular, macerated, and undermined, particularly in the proximal area. The perilesional skin exhibits marked edema and erythema, with desquamation in the proximal portion.	Surgical debridement of necrotic tissue, slough and dry eschar; antimicrobial dressing (i.e., silver containing dressing) or alginate and foam. Evaluate the administration of systemic antibiotics, compression therapy and hyperbaric oxygen therapy.
6	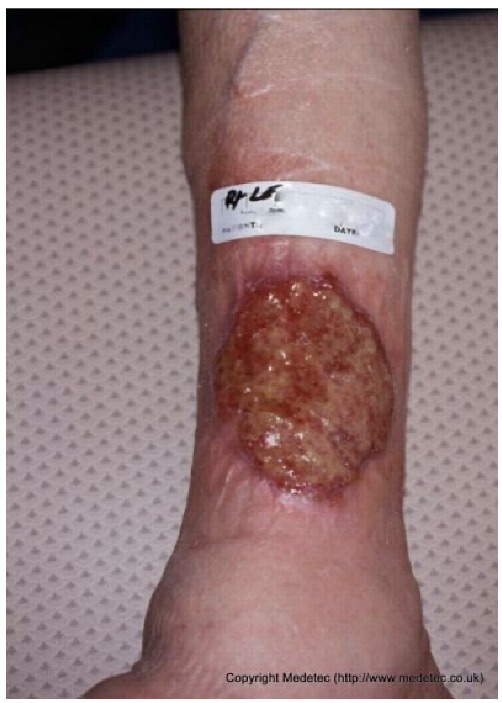	Oval ulcer on the lower leg with a moist granulating base and minimal fibrin, surrounded by erythematous and slightly thickened skin. The wound yet edges are regular and well defined.	Hydrogel dressing to promote autolytic debridement of slough; alginate and foam dressing to maintain moisture balance, combined with graduated compression therapy if venous origin is suspected.	The image shows an ulcer on the lower leg with an irregular, full-thickness appearance. The wound bed is mostly covered by mixed tissue: active, highly vascular granulation tissue (red) interspersed with moderate, loose, pale-yellow slough/fibrin. There is minimal erythema surrounding the wound, and the periwound skin is intact.	Manage the mixed wound bed by applying a calcium alginate or hydrofiber dressing to manage exudate and promote autolytic debridement of the slough. Ensure underlying compression therapy is optimized for this likely venous ulcer.	Oval-shaped ulcer, approximately 8 cm in maximum diameter, of probable venous etiology, located on the distal portion of the right leg. The wound bed is exudative, with granulation tissue and areas covered by a modest amount of slough. Wound margins are regular, surrounded by an erythematous halo. The perilesional skin is edematous, with mild erythema.	Amorphous hydrogel or mechanical abrasion; alginate and polyurethane foam. Evaluate compression therapy.
7	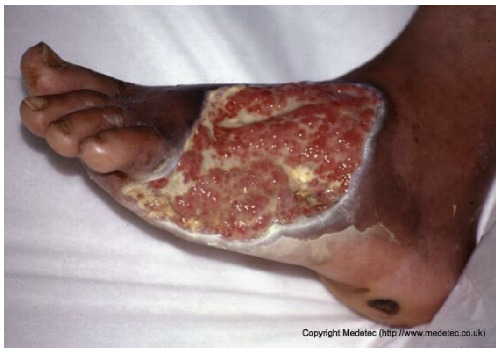	Large dorsal foot ulcer with mixed red granulation tissue and yellow fibrinous areas, partial epithelialization at the edges, and surrounding dusky, macerated skin. Moderate exudate present.	Hydrogel, hydrofiber or alginate dressing with antimicrobial coverage, strict off-loading, and regular debridement to promote granulation and prevent infection.	The image displays a very large, irregular, full-thickness ulcer on the plantar and medial aspect of the foot, extending to the toes. The wound bed is a mix of abundant, friable, pale-red granulation tissue and scattered areas of pale-yellow slough/fibrin. There is significant periwound maceration/peeling, and hyperkeratosis of the surrounding foot skin is evident.	Immediate and strict offloading is mandatory, as this is a likely diabetic foot ulcer. Use a highly absorptive hydrofiber or alginate dressing to manage exudate, combined with an antimicrobial component (e.g., silver) if infection is suspected.	Probable diabetic ulcer involving the plantar, lateral, and dorsal aspects of the left foot, extending from the tarsus to the base of the toes. Full-thickness tissue loss is present, with bright red granulation tissue and multiple areas covered by slough. Wound margins are well-defined and hyperkeratotic. The perilesional skin appears intact.	Amorphous hydrogel and antimicrobial dressing (i.e., silver containing dressing or cadexomer iodine); hydrofiber or alginate dressing and polyurethane foam. Evaluate compression therapy, off-loading, glycemic monitoring and corresponding antidiabetic therapy.
8	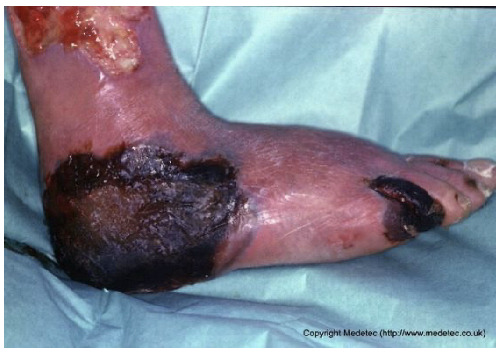	Extensive necrotic ulcer on the lateral and plantar aspects of the foot with black eschar and surrounding erythematous, edematous skin. Distal tissue appears ischemic with early demarcation.	Urgent vascular assessment, dry necrosis management with protective dry dressing, and surgical or enzymatic debridement if perfusion is restored.	The image shows extensive necrotic tissue (black eschar) covering the lateral malleolus and posterior aspect of the foot/heel. The surrounding skin is severely erythematous and dusky/mottled with significant edema, suggesting deep tissue injury, severe ischemia, or gangrene. A separate, smaller eschar is visible near the fifth toe.	Urgent vascular assessment is essential to determine limb viability and potential for revascularization; sharp/surgical debridement of the eschar should only be performed after this assessment. Keep the dry, stable eschar intact (do not debride) and apply a dry, non-adherent dressing until definitive management is planned.	Multifocal ulcers of the right lower limb, likely of mixed arterial, venous, and/or diabetic etiology. At the heel, a large area of demarcated eschar with relatively well-defined margins is present. At the level of the lateral malleolus, a full-thickness irregular ulcer (partially visible) is noted, with an exudative, bleeding, and slough-covered wound bed; margins are violaceous and macerated. Distal eschars are present at the stump of the V toe, the entire IV toe, and the periungual areas of the III and II toes. The perilesional skin is reddish-violet, edematous, and exhibits desquamation in the dorsal region.	Urgent vascular assessment. Dry dressing on the eschars; hydrofiber or alginate dressing and polyurethane foam on the malleolar ulcer.
9	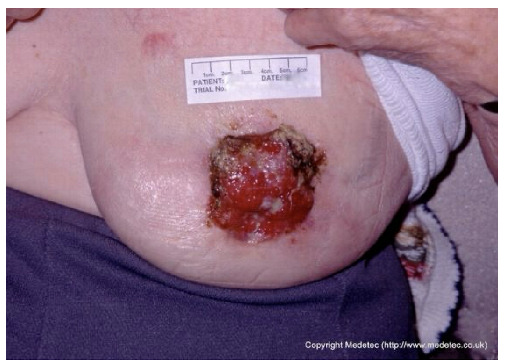	Ulcerated lesion on the lateral breast, approximately 4–5 cm in diameter, with irregular margins, mixed red granulation and yellow-brown necrotic tissue. Surrounding skin shows erythema and mild edema.	Cleanse with saline and apply hydrocolloid or hydrogel dressing to maintain moisture; consider alginate for exudate control and protect periwound skin.	The image shows an irregular, full-thickness wound, approximately 2–3 cm in diameter, located in the infra-mammary/chest wall region. The wound bed is a mix of bright red, friable granulation tissue and peripheral slough/necrotic tissue with a dark, rolled border. The appearance is concerning for a malignant ulcer (e.g., fungating wound) or pyoderma gangrenosum.	Prioritize differential diagnosis/biopsy if malignancy is suspected. Manage symptoms with a non-adherent, lightly absorbent dressing (e.g., silicone foam or hydrogel). Use a topical hemostatic agent (e.g., alginate) if bleeding is a concern.	Probable neoplastic ulcer, round in shape, approximately 5 cm in diameter, located on the left breast at the level of the NAC. The wound bed is exudative, with exuberant granulation tissue, multiple slough-covered areas and small eschars in the superolateral and medial portions. Wound margins are irregular and elevated. The surrounding skin is erythematous and taut.	Punch biopsy of the ulcer. Hydrogel and alginate or hydrofiber dressing and foam.
10	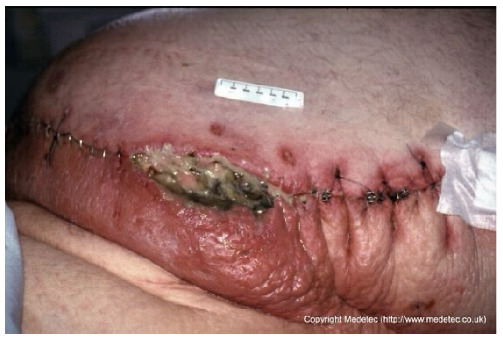	Post-surgical wound dehiscence along a stapled incision line with central necrotic slough and purulent exudate. Surrounding skin is erythematous, edematous, and tense, suggesting local infection or inflammation.	Cleanse with saline, apply antiseptic or silver-impregnated dressing, and consider negative pressure therapy after infection control. Start systemic antibiotics and monitor for surical site infection.	The image shows a poorly healing surgical incision with steel staples, located on the abdomen. A significant portion of the incision line has dehisced, forming a large defect with a base covered by thick, tenacious, dark slough and necrosis. There is marked surrounding erythema, edema, and cellulitis, suggesting severe surgical site infection (SSI) and wound dehiscence.	Urgent surgical intervention is required for debridement of necrotic tissue and source control of the severe infection. Post-debridement, manage the open abdomen with Negative Pressure Wound Therapy (NPWT) or highly absorbent antimicrobial dressings.	Dehiscence of an infected surgical wound, approximately 12 × 3 cm, located in the distal left abdomen. The wound bed is highly exudative, with areas of necrosis, slough, and abundant purulent material; assessment of lesion depth is difficult. Wound margins are irregular, markedly erythematous, and edematous. The perilesional skin is red with intense edema, particularly in the area inferior to the dehiscence and at the sites of sutures and steel staples.	Surgical debridement of necrotic tissue; antimicrobial dressing (i.e., silver containing dressing or cadexomer iodine); hydrofiber dressing and foam. Evaluate the administration of systemic antibiotics, partial or complete sutures and steel staples removal and urgent CT scan.
11	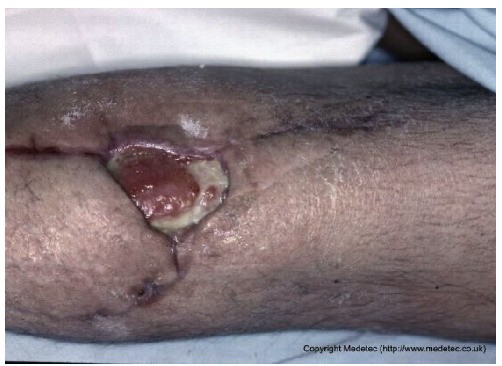	Localized dehiscence over a surgical scar with exposed granulation tissue and yellow fibrinous slough. Margins appear rolled and indurated, with mild surrounding erythema.	Cleanse with saline and apply hydrogel and hydrofiber dressing; protect edges with barrier film.	The image shows a dehisced surgical incision on an extremity, where the wound edges have separated. The resulting defect reveals a wound bed with a mix of fragile, red granulation tissue and pale-yellow slough/fibrin. There is notable serous or purulent exudate and minimal surrounding erythema.	Focus on achieving secondary wound closure: use a hydrofiber or alginate to manage exudate and a hydrogel to autolytically debride the slough. Consider Negative Pressure Wound Therapy (NPWT) if the defect is large and not progressing.	Full-thickness oval-shaped ulcer, likely resulting from partial dehiscence of a surgical wound at the level of the left knee. The wound bed is exudative, partially granulating, with a broad proximal area covered by slough. Wound margins are well-defined but undermined. The perilesional skin shows desquamation, with visible remaining portions of the suture line, which appear hyperpigmented and in the maturation phase. Along the lateral suture, a subcentimetric area of early ulceration is present.	Amorphous hydrogel or selective surgical debridement of slough; hydrofiber and polyurethane foam dressing. Analogous treatment for smaller ulcer.
12	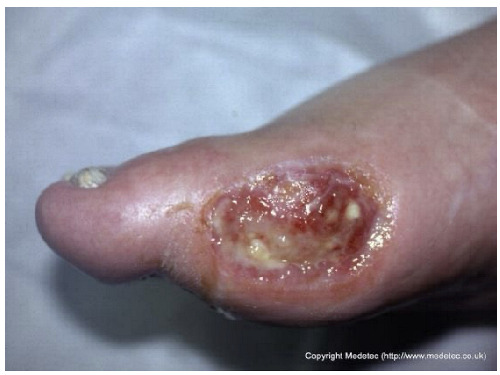	Ulcer on the distal toe with irregular margins, red granulation tissue, and yellow fibrinous exudate. Surrounding skin shows mild maceration and erythema.	Cleanse with saline and apply hydrofiber or alginate dressing; maintain moisture balance and offload pressure from the affected toe.	The image shows an ulcer on the plantar surface of the great toe, likely a diabetic foot ulcer. The wound bed has a mixture of red, moist granulation tissue and moderate amounts of pale-yellow, soft slough. The periwound skin is slightly macerated, and the wound edges appear calloused/hyperkeratotic.	Mandatory strict offloading is required to remove pressure from the ulcer. Debride the hyperkeratotic edges and slough, then apply a moist wound dressing like a hydrogel covered by a foam or hydrocolloid.	Supposed stage III elliptical-shaped pressure ulcer located on the lateral aspect of the first metatarsal head of the right foot. The wound bed is highly exudative and granulating, with evident islands of slough noted primarily in the inferior portion. The margins are well-demarcated and hyperkeratotic superiorly. The perilesional skin is mildly erythematous.	Surgical debridement of islands of slough and hyperkeratotic margins down to a well-vascularized tissue; hydrofiber and polyurethane foam. Evaluate off-loading.
13	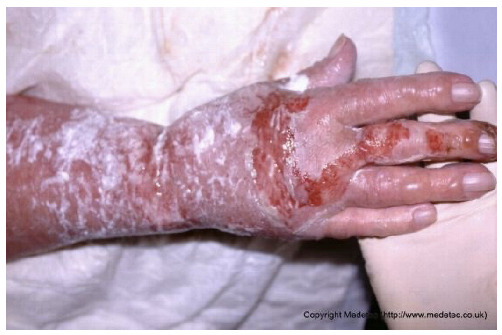	Superficial partial-thickness burn on the dorsal hand and wrist with areas of erythema, serous exudate, and superficial skin loss. No visible necrosis or deep tissue exposure.	Cleanse gently and apply silver sulfadiazine dressing and cover with a non-adherent absorbent dressing; maintain moisture and protect from friction or contamination.	The image shows an injury to the hand and forearm, characterized by a mix of partial-thickness and deeper burns. The forearm is covered by a white, creamy substance (likely a topical cream/ointment), while the dorsum of the hand and fingers show areas of raw, weeping, bright red dermis (superficial/partial thickness) and possible deeper injury on the fingers. There is surrounding erythema and significant edema of the hand.	Management involves thorough wound cleansing and application of a topical antimicrobial agent (e.g., silver sulfadiazine, honey, or a silver dressing) followed by a non-adherent dressing. Control edema with elevation and consider physical therapy to prevent contractures.	Superficial partial-thickness burn to the dorsal aspect of the right hand, involving the metacarpal area and the third digit. The wound bed is minimally exudative, demonstrating alternating areas of granulation and re-epithelialization. The margins are irregular and indistinct. The perilesional skin is edematous and covered with a difficult-to-identify whitish material (likely an emollient or cream).	Gentle wound cleasing; hydrofiber with silver and polyurethane foam. On the perilesional skin: gently remove the cream, petrolatum gauze and sterile gauze.
14	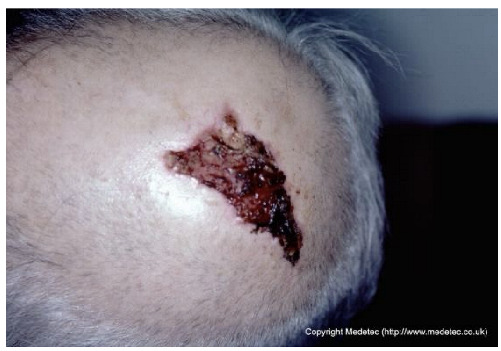	Superficial ulcer on the scalp with mixed red granulation and brown necrotic tissue. Surrounding skin appears mildly erythematous without evident edema.	Cleanse with saline and apply hydrocolloid or hydrogel dressing to maintain a moist environment; protect surrounding skin.	The image displays an irregular, partial-to-full-thickness wound on the scalp/forehead. The wound base is predominantly dark red, bloody, and moist granulation tissue interspersed with adherent dark brown/black eschar/necrotic tissue. There is minimal surrounding erythema, and the wound edges are slightly rolled, raising suspicion for a potential malignancy (e.g., basal or squamous cell carcinoma).	Due to the appearance, obtain an urgent biopsy to rule out malignancy. If non-malignant, use a hydrogel dressing to promote autolytic debridement of the necrotic tissue and cover with a non-adherent foam dressing.	Triangular-shaped ulcer on the left parietal region, with the apex pointing inferolaterally, most likely partial-thickness. The wound base is bleeding and crusted-covered in the inferior portion. The margins are erythematous, serrated, yet well-defined. Neoplastic etiology cannot be excluded. Mild erythema of the surrounding skin.	Punch biopsy of the ulcer; amorphous hydrogel; thin hydrocolloid dressing.
15	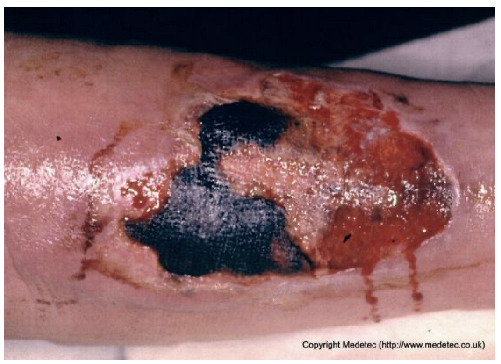	Extensive ulcer on the lower limb with central black eschar and surrounding red granulation tissue. Margins irregular, with moderate exudate and peripheral erythema.	Perform gentle debridement or apply hydrogel or enzymatic dressing to soften necrosis; use hydrofiber and absorbent secondary dressing for exudate control. Monitor for systemic signs.	The image displays a large, irregular ulcer on the lower extremity with a mixed wound bed. Approximately half the bed is covered by hard, black, adherent eschar/necrosis, and the remainder shows bright red, moist granulation tissue. The periwound skin is erythematous and appears macerated/weeping, with serosanguinous exudate visible.	Management requires debridement of the eschar. Apply a hydrogel or enzyme debrider to the necrotic areas and cover with a moisture-retaining, absorptive foam or hydrocolloid dressing to facilitate autolytic debridement and manage exudate.	Extensive, full-thickness ulcer of oval shape on the lower leg, with a proximal eschar and a distal exudative and granulating area. The margins are irregular, yet sharp and well-demarcated. The perilesional skin is edematous and slightly erythematous.	Surgical debridement of the eschar; hydrofiber with silver and polyurethane foam.
16	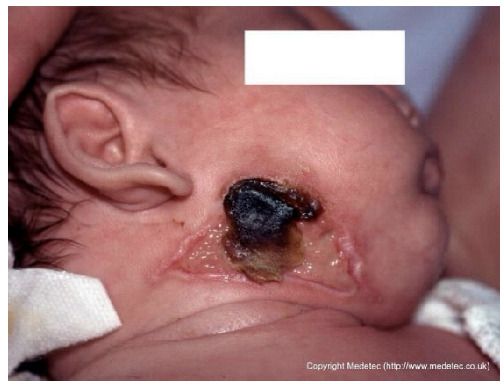	Necrotic lesion with black eschar on the right mandibular and subauricular region, surrounded by erythematous margins and partial epidermal loss. Mild serous exudate present with early demarcation between necrotic and viable tissue.	Cleanse gently with saline, apply hydrogel or enzymatic dressing to promote autolytic debridement, cover with non-adhesive moist dressing and protect surrounding skin with barrier film. Consult a pediatrician to investigate the etiology.	The image shows an ulcerated lesion on the face/neck of an infant, primarily characterized by a central area of black, hard eschar/necrosis. Surrounding the eschar are areas of yellow slough and pale-pink, fragile skin breakdown. The periwound skin is erythematous and intact, and the appearance is concerning for a deep ischemic or infectious process (e.g., Ecthyma gangrenosum).	This wound requires urgent pediatric consultation for underlying etiology and management, likely involving systemic antibiotics and potential surgical debridement if an infectious or ischemic process is confirmed. Topically, maintain a moist environment with a hydrogel under a light, non-occlusive dressing.	Deep ulcer of a trapezoidal shape located on the right mandibular area, consistent with a pressure ulcer. The wound base exhibits abundant slough and eschar in the middle and superior portions. The margins are undulating and well-demarcated. The perilesional skin appears erythematous and edematous.	Amorphic hydrogel on eschar and slough; polyurethane foam. Evaluate pressure-relieving devices and alternative patient repositioning.
17	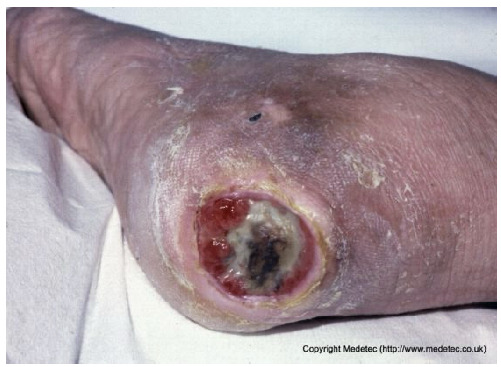	Circular ulcer on the heel with necrotic tissue at the base, yellow slough, and peripheral granulation. Surrounding skin shows erythema, dryness, and scaling, consistent with a chronic pressure ulcer.	Debridement of necrotic tissue followed by hydrocolloid or hydrogel dressing; maintain moist wound environment and offload pressure.	The image shows a deep, circular ulcer on the heel with a well-defined, hyperkeratotic/calloused border, characteristic of a neuropathic/pressure ulcer. The wound base contains a mix of red granulation tissue, yellow slough, and a small amount of central black eschar. The surrounding skin is dry and hyperkeratotic.	Implement immediate and strict offloading for the heel. Debride the calloused edges and necrotic/sloughy center. Apply a hydrogel to the base under an absorptive foam dressing for autolytic debridement and moisture balance.	Plausible stage III or stage IV pressure ulcer, rounded shaped, on the posterior aspect of the right heel. The wound bed is exudative, with peripheral and distally exuberant granulation tissue and a central area of slough and necrosis. The margins are well-defined, raised, and exhibit a callused border. The perilesional skin is atrophic with generalized desquamation.	Amorphic hydrogel on the necrosis and surgical debridement of the callused border; alginate and polyurethane foam. Use pressure-relieving devices and compression therapy.
18	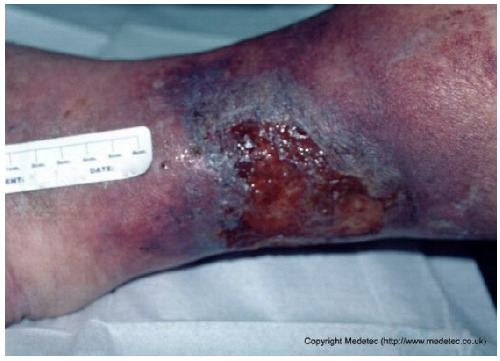	Irregular superficial ulcer on the lower leg with a moist red base and partial epithelial loss. Surrounding skin shows hyperpigmentation, edema, and signs of chronic venous stasis.	Apply hydrocolloid and foam dressing and initiate multilayer compression therapy; elevate limb to reduce venous stasis.	The image shows a shallow, irregular ulcer located on the medial aspect of the ankle/lower leg, surrounded by pronounced hyperpigmentation and induration, characteristic of lipodermatosclerosis. The wound bed is mainly covered by clean, red, moist granulation tissue with low exudate and minimal slough.	Strictly maintain compression therapy for the underlying venous insufficiency. Apply a non-adherent contact layer or a thin hydrocolloid dressing to protect the granulation tissue and promote healing with minimal disruption.	Superficial ulcer of an irregularly triangular shape with a maximum length of approximately 5 cm, located on the postero-medial aspect of the right distal lower leg. Plausible venous etiology. The wound bed is exudative, with markedly edematous granulation tissue interspersed with islands of slough. The margins are irregular, violaceous and macerated. The perilesional skin is moderately edematous and darkly pigmented, highly suggestive of venous insufficiency.	Gentle surgical debridement of islands of slough; hydrofiber and polyurethane foam. Compression therapy with zinc oxide bandage.
19	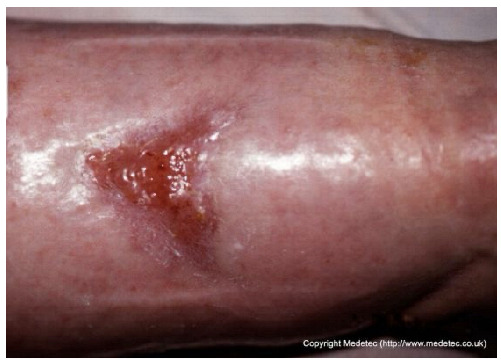	Small superficial ulcer with partial epithelial loss and granulation tissue at the base. Surrounding skin appears shiny, taut, and mildly erythematous, suggestive of venous insufficiency.	Use hydrocolloid or foam dressing to maintain moisture and apply graduated compression therapy to address venous insufficiency.	The image displays a small, shallow, triangular ulcer on the lower leg. The wound base is primarily covered by bright red, healthy-appearing granulation tissue with a small amount of peripheral yellow slough/fibrin. The surrounding skin is erythematous, edematous, and appears glossy/taut, suggesting underlying venous disease or inflammation.	If a venous ulcer is suspected, maintain compression therapy. Apply a hydrocolloid or thin foam dressing to protect the granulation tissue and manage low exudate, or a hydrogel if slough needs autolytic debridement.	Small, triangular-shaped superficial ulcer, likely located on the anterior aspect of the lower leg. The wound bed is granulating and mildly edematous, with small areas of fibrin in the most proximal portion. The margins are irregular and sloping, showing signs of initial re-epithelialization. The surrounding skin is diffusely edematous.	Gentle surgical debridement of the small areas of fibrin; thin hydrocolloid dressing; compression therapy.
20	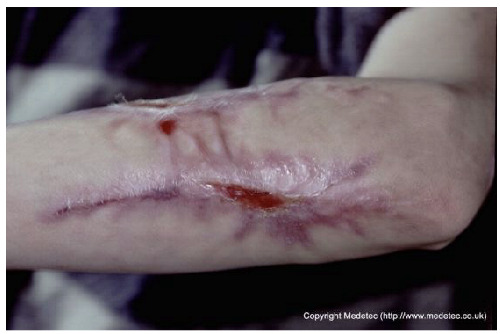	Irregular hypertrophic scar on the forearm with areas of epithelial breakdown and partial ulceration. The wound bed appears red and moist, surrounded by fibrotic, shiny scar tissue with mild local erythema. No signs of active purulent exudate are visible.	Use a non-adherent silicone or hydrogel dressing to maintain moisture balance and protect fragile tissue; consider compression and scar management therapy.	The image displays a mature, pale, slightly raised, hyper-pigmented linear scar on the forearm, suggesting a healed previous injury or incision. There are two areas of superficial wound dehiscence/breakdown along the scar line, exposing small amounts of bright red, moist granulation tissue with minimal exudate. The surrounding skin shows post-inflammatory hyperpigmentation.	Management involves minimizing tension/movement across the area. Apply a thin hydrocolloid or transparent film to protect the small areas of granulation tissue and facilitate re-epithelialization.	Superficial ulcer located along a hypertrophic and hyperpigmented scar, plausibly from a burn injury, situated on the dorsal aspect of the left forearm. The wound bed is granulating with modest peripheral fibrin. The margins are violaceous and fibrotic. Medially and more distally, there is a subcentimeter ulcer with analogous characteristics.	On main and smaller ulcer: surgical debridement of the fibrotic margins; thin hydrocolloid dressing; compression therapy. Evaluate scar management therapy.

**Table 2:** Visual concordance matrix comparing AI and surgeons’ evaluations. Each case was assessed by ChatGPT and Gemini for wound description and management. The color scale indicates the agreement level with the surgeons’ consensus: Red = discordant (inaccurate or unsafe response); Yellow = partially concordant (incomplete or partially correct); Green = fully concordant (clinically accurate and aligned with expert consensus).

Case ID	Wound Description ChatGPT	Management ChatGPT	Wound Description Gemini	Management Gemini
1				
2				
3				
4				
5				
6				
7				
8				
9				
10				
11				
12				
13				
14				
15				
16				
17				
18				
19				
20				

**Table 3:** Quantitative performance metrics of ChatGPT and Gemini compared with the expert panel.

Assessment	Model	Mean Score (SD)	Score Distribution (0/1/2)	Perfect Agreement n (%)	95% CI	*p*-Value (vs. 50%)	*p*-Value (vs. Gemini)
Wound Description	ChatGPT	1.50 (0.51)	0/10/10	10 (50.0%)	29.9–70.1%	1.000	
	Gemini	1.55 (0.69)	2/5/13	13 (65.0%)	43.3–81.9%	0.263	0.508
	*Inter-model Agreement*			Cohen’s κ = 0.000			
Management	ChatGPT	1.60 (0.50)	0/8/12	12 (60.0%)	38.7–78.1%	0.503	
	Gemini	1.70 (0.57)	1/4/15	15 (75.0%)	53.1–88.8%	0.041	0.453
	*Inter-model Agreement*			Cohen’s κ = 0.255			
Overall Performance	ChatGPT	3.10 (0.64)	—	5 (25.0%)	—	—	
	Gemini	3.25 (0.97)	—	9 (45.0%)	—	—	0.388

## Data Availability

No new data sere created or analyzed in this study.

## Abbreviations

The following abbreviations are used in this manuscript:

LLMs	Large Language Models
TIME	Tissue, Infection/Inflammation, Moisture balance, Edge
TIME CDST	TIME Clinical Decision Support Tool
TIME-H	TIME Health model
NPWT	Negative Pressure Wound Therapy
AI	Artificial Intelligence
MRI	Magnetic Resonance Imaging
USD	United States Dollar
